# A performance comparison between fluorescent immunoassay and immunochromatography for rapid dengue detection in clinical specimens

**DOI:** 10.1038/s41598-022-21581-x

**Published:** 2022-10-14

**Authors:** Kriangsak Ruchusatsawat, Tanawat Benjamungkalarak, Naruphong Phunikom, Husneeyah Vateh, Ekasit Kowitdamrong, Jongkonnee Wongpiyabovorn, Siwaporn Boonyasuppayakorn

**Affiliations:** 1 National Institute of Health, Department of Medical Sciences, Ministry of Public Health, Nonthaburi, Thailand; 2 Center of Excellence in Applied Medical Virology, Department of Microbiology, Faculty of Medicine, Chulalongkorn University, Bangkok, Thailand; 3 Center of Excellence in Immunology and Immune Mediated Diseases, Division of Immunology, Department of Microbiology, Faculty of Medicine, Chulalongkorn University, Bangkok, Thailand

**Keywords:** Immunology, Microbiology, Molecular biology, Medical research

## Abstract

Dengue virus (DENV 1–4) infection has been a global health threat where no specific treatment is currently available. Therefore, a rapid and accurate diagnosis is critical for an appropriate management as it could reduce the burden of severe clinical manifestation. Currently, dengue immunochromatography (IC) is commonly used to primarily differentiate acute febrile illnesses. Fluorescent immunoassay (FIA) utilized a highly sensitive detection system and claimed 70–100% sensitivity and 83.5–91.7% specificity for dengue infection in a preliminary report. This report recruited samples with acute febrile illnesses sent for dengue screening and tested IC and FIA in parallel. The performance of both tests was verified by a definitive diagnosis retrieved from combinatorial reverse transcription-quantitative polymerase chain reaction and enzyme-linked immunosorbent assay (ELISA) for IgM and IgG confirmation tests. Results showed that the viral nonstructural protein (NS1) performance of FIA was slightly higher than IC with the sensitivity, specificity, PPV, NPV, agreement, kappa, and its standard error at 79.11, 92.28, 86.81, 87.31, 352 (87.13%), 0.725 ± 0.035, respectively; whereas those of the IC were at 76.58, 92.28, 86.43, 85.98, 348 (86.14%), 0.703 ± 0.037, respectively. Moreover, the IgM and IgG performance of FIA had higher specificity, PPV, and agreement than the IgM IC performance, suggesting that the FIA was more specific but less sensitive for antibody detection. No correlation was observed in IgM and IgG levels of ELISA and FIA assays. In conclusion, the FIA and IC were highly sensitive, specific, and substantially agreed in NS1 detection but moderately agreed in IgM and IgG detection.

## Introduction

Dengue virus (DENV 1–4) infection has been a global health threat, with at least two billion people being at risk^1^. In Thailand, the cumulative data from 1958 by the Department of disease control approximates the 50,000 annual cases, and the outbreak usually occurs once every 2–3 years^2^. Recently, the 2019 outbreak accounted for 130,705 cases and 142 deaths^2^. The virus is a member of the family *Flaviviridae*, consisting of an RNA genome and a lipid envelope^3^. The severe clinical manifestation caused by the secondary heterotypic infection includes high graded fever, severe myalgia, thrombocytopenia, hemoconcentration from plasma leakage, hypovolemic shock, and death^4^. Unfortunately, the only commercial vaccine is limited to individuals with confirmed previous infection in the endemic area^5^. Moreover, no specific drug is currently available^6^. Moreover, Thailand is endemic to other mosquito-borne viruses, in which the DENV1 and chikungunya (CHIKV) are actively circulating during 2017–2019 with > 10,000 cases per year. Therefore, rapid detection still plays a critical role in appropriately managing dengue patients.

Point-of-care dengue diagnostic tests generally use the rapid immunochromatography platform to detect dengue-specific nonstructural protein 1 (NS1). The sensitivity and specificity of NS1 RDTs evaluated by WHO/TDR/PDVI laboratory network were 40–75%, and 93–100%, whereas those of the IgM and IgG were 53–87.5% and 46–100%, respectively^7^. The false negative NS1 detection is usually limited to the sensitivity of a colorimetric reporter system and a manual inspection. The false positive is generally caused by cross-reactivity of antigen–antibody detection, especially in IgG. However, a recently developed fluorescent immunoassay (FIA) claimed to increase the sensitivity by using fluorescent-labeled antibodies, and the automated detection system claimed to reduce manual bias. Moreover, the preliminary results suggested that the sensitivity of FIA-DENV NS1Ag, IgM, and IgG was 100%, 100%, and 70%, whereas the specificity of FIA-DENV NS1Ag, IgM, and IgG was 87.5%, 91.7%, and 83.5%, respectively^8^. This article focused on implementing the FIA on a large scale and compared its performance with the currently used rapid immunochromatography.

## Objective

To evaluate the performance of Standard F Fluorescence Immunoassay (FIA) for dengue NS1, IgM, and IgG, produced by SD. Biosensor Inc., Suwon, South Korea, compared the results with the SD Bioline Dengue Duo Immunochromatography (IC) for dengue NS1, IgM, and IgG by SD. Biosensor Inc., Suwon, South Korea.

## Results

### Sample recruitment

**Figure 1:**
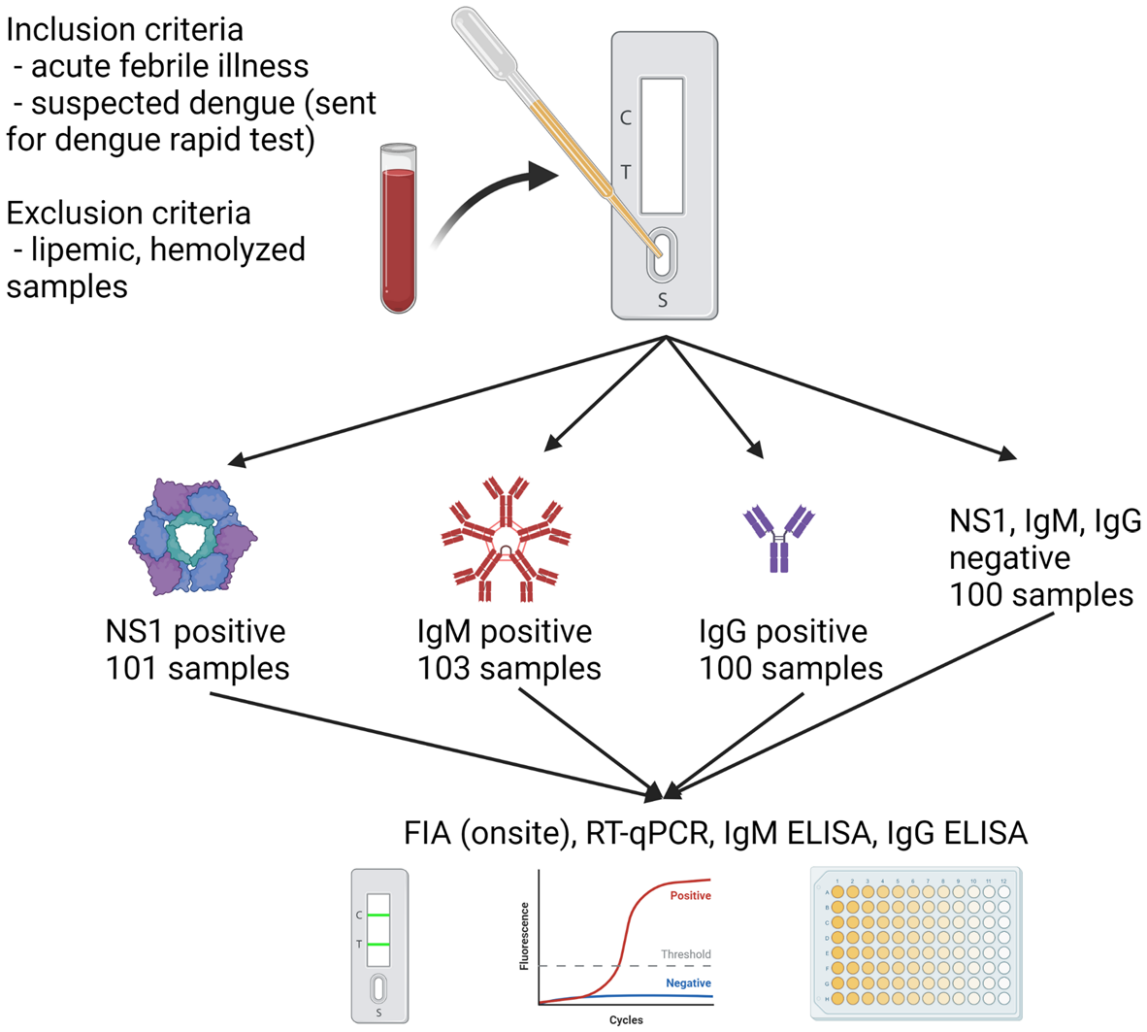
Flowchart of sample collection, categorization, and series of analytical tests, created by Biorender.

All 404 plasma samples were categorized into four groups according to their immunochromatography results (Fig. 1) and the cluster sampling was performed. Briefly, the samples sent for rapid dengue immunochromatography at the King Chulalongkorn Memorial Hospital were prospectively collected during September 5, 2019 to September 4, 2020 and categorized into 101 NS1 positives, 103 IgM positives, 100 IgG positives, and 100 NS1, IgM, and IgG negative samples. Dual positive results were randomly assigned into any of the positive groups. The fluorescent immunoassay was performed at KCMH, whereas the RT-qPCR and IgM, and IgG ELISA^9^ were done at arbovirus unit, Thai National Institute of Health, Department of Medical Sciences, Thailand. The definitive diagnosis was made from the combinatorial results of RT-qPCR and ELISA^9,10^.

### Demographic data

The patients’ demographic data demonstrated that the major population were gender-balanced adult (Table 1). The average (mean ± SD) date of specimen collection after fever onset was 3.30 ± 2.84 days. The RT-qPCR method characterized the results to DENV1 (18.06%, 73/404 cases), DENV2 (9.65%, 39/404 cases), DENV3 (1.98%, 8/404 cases), DENV4 (0.74%, 3/404 cases), CHIKV (6.44%, 26/404 cases), not detected (60.89%, 246/404 cases). Nine samples with a volume less than 100 micromolar were categorized as inadequate for RNA extraction (2.23%, 9/404 cases). Of which, four out of nine inadequate samples were successfully diagnosed as probable dengue infection by IgM ELISA at ≥ 40 units/ml. Therefore, the remaining five samples were characterized as not determined. The additional 31 cases were identified as probable dengue infections based on the criteria of IgM ELISA ≥ 40 units/ml, IgG ELISA at any units/ml; and less than seven days of fever onset^9^; resulting the total number of IgM-positive cases at 35. Moreover, two cases were diagnosed as other flaviviral infections from IgG ELISA levels of ≥ 100 units/ml and IgM ELISA level of < 40 units/ml. The remaining 213 samples were identified as acute febrile illness from other causes or unspecified. Other diagnoses included influenza, other viral infection and bacterial infection of the respiratory tract, non-infectious hyper inflammation, and unspecified fever. Collectively, a total of 158 cases were dengue-confirmed by RT-qPCR and ELISA results. Moreover, 26 chikungunya infections and 2 other flaviviral infections were diagnosed from RT-qPCR and ELISA, respectively. Based on the epidemiological data of 2019, the other flaviviral infections were likely Zika virus infections.

**Table 1:** Demographic data of the patients.

Patients’ characteristics		Remarks
Gender: male	50.12%	
**Age (years)**		
< 18	14.60% (57/404)	
18 and above	85.40% (347/404)	
Median date of fever onset [IQR]	3 [2, 4]	
**Laboratory diagnosis (method)**		
Dengue virus infection	39.11% (158/404)	
DENV1 (RT-qPCR)	18.06% (73/404)	
DENV2 (RT-qPCR)	9.65% (39/404)	
DENV3 (RT-qPCR)	1.98% (8/404)	
DENV4 (RT-qPCR)	0.74% (3/404)	
Probable dengue infection (ELISA)	8.66% (35/404)	ELISA IgM ≥ 40 units, IgG any units; fever onset < 7 days
Other diagnoses	60.89% (246/404)	
CHIKV (RT-qPCR)	6.43% (26/404)	
Other flaviviral infection (ELISA)	0.50% (2/404)	ELISA IgM < 40 units, IgG ≥ 100 units
Acute febrile illness (e.g. other viral or bacterial infections, or unspecified causes)	52.72% (213/404)	
Not determined	1.23% (5/404)	Inadequate samples for RT-qPCR, ELISA IgM < 40 units, ELISA IgG < 100 units)

### Performance comparison between two rapid tests

The performance of two rapid tests were compared with the definitive diagnosis made by combinatorial RT-qPCR and ELISA results (Table 2). Parameters in this study were sensitivity, specificity, positive predictive value, negative predictive value, agreement, kappa index, and their standard errors. In antigen detection, the sensitivity of FIA was slightly higher than that of IC at 79.11% and 76.58%, respectively, while the specificities of both rapid tests were equal at 92.28%. Moreover, the agreement of FIA was slightly superior to those of IC at 87.13% and 86.14%, respectively. Both FIA and IC rapid tests displayed substantial agreement in kappa analysis at 0.725 ± 0.035 and 0.703 ± 0.037, respectively. The parameters of RT-qPCR were also calculated as a reference test for rapid antigen detection. Since 123 out of 158 dengue-confirmed cases were diagnosed by RT-qPCR, perfect agreement (> 0.8) between the RT-qPCR and definitive diagnosis was expected. To conclude, the NS1 FIA was slightly higher than NS1 IC in sensitivity, PPV, NPV, agreement, and kappa index.

**Table 2:** Sensitivity, specificity, PPV and NPV of FIA and IC to detect NS1, IgM and IgG compared with the confirmed cases.

	Sensitivity	Specificity	PPV	NPV	Agreement (n, %)	Kappa ± SE
**RT-qPCR**						
Genome	77.85	100.00	100.00	87.54	369 (91.34%)	0.811 ± 0.030
**ELISA**						
IgM and IgG	41.14	97.97	92.86	72.16	306 (75.74%)	0.434 ± 0.042
**FIA**						
NS1	79.11	92.28	86.81	87.31	352 (87.13%)	0.725 ± 0.035
IgM and IgG	40.51	89.84	71.91	70.16	285 (70.54%)	0.329 ± 0.046
**IC**						
NS1	76.58	92.28	86.43	85.98	348 (86.14%)	0.703 ± 0.037
IgM	53.80	80.89	64.39	73.16	284 (70.30%)	0.357 ± 0.048
IgG	43.04	53.66	37.36	59.46	200 (49.50%)	− 0.032 ± 0.049

*PPV* positive predictive value, *NPV* negative predictive value.

The value of kappa index was interpreted according to the following scale; < 0: poor agreement, 0–0.2: slight agreement, 0.21–0.40: fair agreement, 0.41–0.60: moderate agreement, 0.61–0.80: substantial agreement, 0.81–1.00: perfect agreement.

Similarly, the antibody detection of two rapid tests were analyzed by the parameters previously described. In this case, the result from IgM and IgG ELISA was used as a reference. In the ELISA and FIA, IgM and IgG levels were interpreted together and yielded a single positive or negative result. On the contrary, the IC results were manually read and separately reported (Table 2). The sensitivity of ELISA, FIA, IgM, and IgG IC tests were 41.14, 40.51, 53.80, and 43.04, respectively. The results suggested the overall poor sensitivity of the antibody tests to detect acute dengue infection. However, the specificity of ELISA was the highest at 97.97%, followed by those of FIA at 89.84%, IgM IC at 80.89%, and IgG IC at 53.66%, respectively. Similarly, the PPV, NPV, and agreement of the ELISA was the highest, followed by FIA, IgM IC, and IgG IC, respectively. The kappa index was moderate in ELISA but was fair in FIA and IgM IC. Interestingly, the PPV and NPV of FIA were 71.91% and 70.16%, respectively, suggesting the potential value in clinical use. Moreover, the IgG IC results showed the lowest performance in all parameters. To conclude, the overall performance of IgM and IgG FIA results was higher than that of the IC, but still lower than that of the ELISA.

### Correlation between the IgM and IgG levels from FIA and ELISA

**Figure 2:**
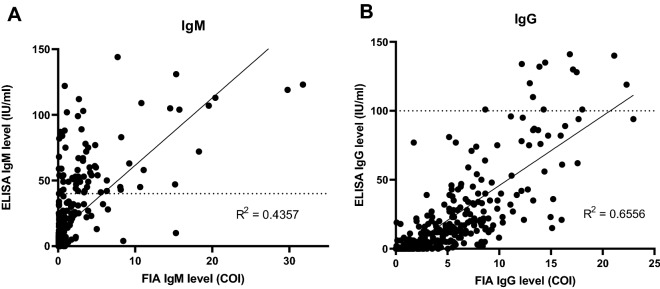
(**A**) IgM and (**B**) IgG levels from the FIA (COI) and ELISA (IU/ml) were plotted. The dot lines represented IgM and IgG ELISA cut-off values at 40 and 100 IU/ml. The cut-off index (COI) ratio between test and control bands on each FIA is referred to signal strength^11^. The coefficient of determination (R^2^) was shown, created by GraphPad Prism.

The IgM and IgG levels from FIA and ELISA tests were tested for the possible correlation. The IgM and IgG levels from the two tests showed no correlation with the *R*^2^ at 0.4357, and 0.6556 (Fig. 2), respectively, likely because of the different measurement units. The cut-off index (COI) of FIA was a ratio from the background signal, whereas the IU/ml of ELISA was an absolute value subtracted by the background signal. Moreover, we compared qualitative readouts of FIA to ELISA assuming the ELISA was a standard reference method for antibody readouts. The sensitivity, specificity, PPV, NPV, agreement and kappa index (± SE) of FIA were 76.39%, 89.76%, 61.80%, 94.60%, 353 (87.38%), 0.605 ± 0.050, respectively. Interestingly, the NPV between tests was > 90% and the kappa index was between moderate and substantial agreement. In contrast, the sensitivity and positive predictive value between tests were moderately correlated. Therefore, the FIA negative results could represent the ELISA negative results with > 90% confidence, whereas the positive FIA results could not.

## Discussion

Rapid dengue detection, especially during acute infection, is critical for confirming the clinical diagnosis. Early and appropriate treatment could significantly prevent the progression to severe diseases such as hypovolemic shock, plasma leakage, bleeding, or multiple organ failure. The currently used rapid immunochromatography showed moderate sensitivity and high specificity. This work reported the NS1 FIA sensitivity, specificity, PPV, NPV, agreement, kappa, and its standard error at 79.11%, 92.28%, 86.81%, 87.31%, 352 (87.13%), 0.725 ± 0.035, respectively, which were slightly superior to those of the IC (Table 2). Therefore, the FIA NS1s' performance was more efficient in detecting and excluding acute dengue infections. Moreover, both rapid assays equally detected false positive CHIKV infections at 7.69% (2/26). Moreover, we found that all false positive cases of FIA and IC were perfectly matched. Since the FIA and IC assays were manufactured by the same company, the results suggested that the NS1 antibody could originate from the exact clone and detect the same epitope. The cross-reactivity could occur to any CHIKV proteins. However, we would speculate the heavily glycosylated spike of CHIKV as DENV NS1 also has an N-linked glycosylation character.

The IgM and IgG FIA sensitivity, specificity, PPV, NPV, agreement, kappa, and its standard error were at 40.51%, 89.84%, 71.91%, 70.16%, 285 (70.54%), 0.329 ± 0.046, respectively. The performance of the FIA was superior to IgM IC in specificity, PPV, and agreement. The IgM IC was more sensitive but less specific than FIA for the antibody detection. Moreover, the distribution patterns of IgM and IgG levels of ELISA and FIA were poorly aligned (Fig. 2A,B), consistent with the low to moderate sensitivity of the FIA. Additionally, the qualitative results of FIA and ELISA were compared and only the NPV was > 90%. The kappa index was borderline between moderate and substantial agreement. ELISA and FIA were performed under different sets of antibodies, reagents, and protocols; therefore, any of those factors could contribute to the different results and interpretations. However, an antibody-based detection (ELISA, FIA, IC) from a single specimen can provide only a presumptive diagnosis. In this case, the convalescent sera were not available for a definitive diagnosis due to the patient care management protocol (mostly out-patient cases).

Next, the laboratory management issues were taken into consideration. The approximate cost per test of NS1/IgM/IgG combo FIA and IC were quoted at 400 THB (12.28 USD) and 350 THB (10.75 USD), respectively (MP biomedicals, Thailand). The FIA variable and fixed costs was slightly higher than that of the IC which could be result from the machine-readout system. The machine read system has an advantage in eliminating the interpersonal variation, but its disadvantage is the limited number (1–24) of assays per run. Both assays require the same specimen type, volume, and turnaround time. Therefore, a similar management system can be applied to both assays.

In conclusion, the FIA was approximately 90% sensitive and specific in NS1 detection and 90% specific, but not sensitive, in IgG/M detection. The IC was more than 90% sensitive and specific in NS1 detection, but neither sensitive nor specific for IgM and IgG detection.

## Materials and methods

### Specimen collection and experimental workflow

The study was done at the Virology unit, King Chulalongkorn Memorial Hospital and arbovirus unit, Thai National Institute of Health, Department of Medical Sciences, Thailand from September 5, 2019 to September 4, 2020 with IRB and IBC approvals (COA 964/2019, IRB 415/62, MDCU-IBC011/2019) from Ethical and Biosafety Review boards, Faculty of Medicine, Chulalongkorn University. All methods were performed in accordance with the relevant guidelines and regulations. The Ethical and Biosafety Review boards, Faculty of Medicine, Chulalongkorn University, waived the requirement for informed consent, since leftovers specimens from diagnosis that are not individually identifiable are under expedited review. All plasma samples and their rapid SD Bioline Dengue Duo Immunochromatography results were courtesy of the Virology unit, King Chulalongkorn Memorial Hospital. The leftover samples were recruited and categorized according to inclusion and exclusion criteria (Fig. 1). The inclusion criteria were acute febrile illness with clinical suspicion for dengue infection (e.g. high-grade fever (> 38.5 °C, not fully return to baseline after taking acetaminophen), myalgia, history of dengue infection in neighbors or family members, positive tourniquet test or petechiae, history of bleeding or menorrhagia, hepatic enlargement, sign of impending shock, etc.). The blood samples were generally sent for hematological workup and dengue rapid test simultaneously. Leftover samples underwent cluster samplings. Briefly, 404 samples were prospectively collected and categorized into 101 NS1 positives, 103 IgM positives, 100 IgG positives, and 100 NS1, IgM, and IgG negative samples. The fluorescent immunoassay was performed onsite within 24 h after the IC according to the manufacturers’ instructions. Samples were then stored at − 70 °C for subsequent confirmation with RT-qPCR (abTES DEN/CHIKU 5 qPCR II Kit, AITbiotech Pte Ltd, Singapore), IgM, and IgG ELISA^9^, respectively at arbovirus unit, Thai National Institute of Health, Department of Medical Sciences, Thailand. The definitive diagnosis was made from the combinatorial results of RT-qPCR and ELISA^9,10^.

### Immunochromatography (IC)

Plasma samples sent for MV079 dengue IgG/IgM/NS1 (rapid) service were collected in a 10.8 mg K2EDTA, blood collection tube (6 ml) (BD Vacutainer, Franklin Lakes, NJ, USA). The samples were centrifuged at 500*g* for 10 min at room temperature, and plasma was collected to perform rapid immunochromatography according to the manufacturers’ protocol (SD Bioline Dengue Duo Immunochromatography, SD BIOSENSOR Inc., Suwon, South Korea). The plasma volume required for NS1 and IgG/IgM ICs were 100 and 10 µl, respectively. The IC cassettes were incubated for 15–20 min at room temperature before the results were read manually and subsequently reported onto the HIS/LIS system.

### Fluorescent immunoassay (FIA)

The plasma samples at 100 and 10 µl were analyzed by Fluorimetric SD-Biosensor-STANDARD-F-Dengue-RDT for dengue NS1, and IgG/IgM, respectively (SD BIOSENSOR Inc., Suwon, South Korea) according to the manufacturers’ instructions. The FIA cassettes were incubated for 15–20 min at room temperature before the results were read under the STANDARD F200 Analyzer (SD Biosensor Inc., Suwon, South Korea). The leftover samples were then stored at − 70 °C and transported on ice for subsequent confirmation tests at the arbovirus unit, NIH, Thailand.

### Genome detection

The frozen samples were thawed on ice, and the 140 µl volume was taken to RNA extraction using the QIAamp@ Viral RNA Mini kit (Qiagen, Hilden, Germany). The RT-qPCR was performed using the commercial abTES DEN/CHIKU 5 qPCR II Kit (AITbiotech Pte Ltd, Singapore) and the 7500 Realtime PCR System (Applied Biosystems, Thermo Fisher Scientific. Waltham, Massachusetts, USA). Chikungunya virus was circulating during the study period.

### ELISA

The in-house IgM and IgG ELISAs utilized the anti-human IgM or anti-human IgG to coat the plate. First, the sample volume at 10 µl was incubated within the wells for 60 min. After specimen incubation, the combined DENV1–4 antigens from C6/36 cells in protein-free media were added, followed by the addition of the monoclonal 4G2, anti-mouse HRP, TMB substrate, and HCl, respectively. Then, the plates were read at the *A*_450nm_ using a Microplate ELX-800 ELISA reader (BioTek, Winooski, VT, USA).

### Statistical analysis

The sensitivity, specificity, positive predictive value (PPV), negative predictive value (NPV) and accuracy of FIA and IC were analyzed according to the definitive diagnosis drawn by RT-qPCR and ELISA results. Agreement between rapid tests (FIA and IC) and the definitive diagnosis was assessed by kappa statistics (GraphPad Prism, La Jolla, CA, USA). The correlation of IgM and IgG levels between FIA and ELISA was analyzed using simple linear regression (GraphPad Prism, La Jolla, CA, USA).

## Data Availability

All supporting data is available upon request. Please contact Siwaporn.b@chula.ac.th.

## References

[1] Bhatt S (2013). The global distribution and burden of dengue. Nature.

[2] Bureau of Epidemiology, MPH. *National Disease Surveillance *(*Report 506*). http://doe.moph.go.th/surdata/disease.php?ds=66 (2022).

[3] Barrows NJ (2018). Biochemistry and molecular biology of flaviviruses. Chem. Rev..

[4] Martina BE, Koraka P, Osterhaus AD (2009). Dengue virus pathogenesis: An integrated view. Clin. Microbiol. Rev..

[5] Paz-Bailey G (2021). Dengue vaccine: Recommendations of the Advisory Committee on Immunization Practices, United States, 2021. MMWR Recomm. Rep..

[6] Wilder-Smith A (2020). Dengue vaccine development: Status and future. Bundesgesundheitsblatt Gesundheitsforschung Gesundheitsschutz.

[7] Hunsperger EA (2014). Evaluation of commercially available diagnostic tests for the detection of dengue virus NS1 antigen and anti-dengue virus IgM antibody. PLoS Negl. Trop. Dis..

[8] Zammarchi L (2019). Evaluation of a new rapid fluorescence immunoassay for the diagnosis of dengue and Zika virus infection. J. Clin. Virol..

[9] Innis BL (1989). An enzyme-linked immunosorbent assay to characterize dengue infections where dengue and Japanese encephalitis co-circulate. Am. J. Trop. Med. Hyg..

[10] Rodriguez-Manzano J (2018). Improving dengue diagnostics and management through innovative technology. Curr. Infect. Dis. Rep..

[11] Kahn M (2021). Performance of antigen testing for diagnosis of COVID-19: A direct comparison of a lateral flow device to nucleic acid amplification based tests. BMC Infect. Dis..

